# Scaling Up Global Health Interventions: A Proposed Framework for Success

**DOI:** 10.1371/journal.pmed.1001049

**Published:** 2011-06-28

**Authors:** Gavin Yamey

**Affiliations:** Evidence-to-Policy Initiative, Global Health Group, San Francisco, California, United States of America

## Abstract

Drawing upon interviews with experts and a review of the literature, Gavin Yamey proposes a new framework for scaling up global health interventions.

**Summary Points**

- The rise in international aid to fund large-scale global health programs over the last decade has catalyzed interest in improving the science of scale-up.
- This Essay draws upon key themes in the emerging science of large-scale change in global health to propose a framework for explaining successful scale-up.
- Success factors for scaling up were identified from interviews with implementation experts and from the published literature.
- These factors include the following: choosing a simple intervention widely agreed to be valuable, strong leadership and governance, active engagement of a range of implementers and of the target community, tailoring the scale-up approach to the local situation, and incorporating research into implementation.

The adoption of the Millennium Development Goals—coupled with the recent rise in international aid for health—has catalyzed interest in improving the science of scale-up [1]. Global health researchers have realized the need for “a quantitative, scientific framework to guide health-care scale-up in developing countries” [2], a need that has begun to draw the attention of donors [3]. Low- and middle-income countries (LMICs) have begun to study effective ways to deliver proven interventions at scale [4],[5]. Thus, there are promising signs that a “science of large-scale change in global health” is emerging [5].

In this Essay, I draw upon key themes in this emerging science to propose a framework for explaining successful scale-up. This framework is aimed at planners of scale-up processes to use in thinking about strategies for implementing a new program, policy, or intervention to scale.

The term “scaling up” is now widely used in the public health literature, but there is no agreed definition. The term is primarily used, say Mangham and Hanson, to describe “the ambition or process of expanding the coverage of health interventions” [6], a working definition that I use in this article.

## Approach to Developing a Framework

The initial ideas for this framework were derived from a 2009 fellowship in global health reporting, which took me to East Africa to report on scaling up insecticide-treated bed nets [7], and drugs for controlling intestinal worms and malaria [8],[9]. I built upon this field reporting by reviewing the relevant literature on scaling up global health interventions in LMICs and by interviewing experts in large-scale change in global health. Text S1 gives further details of the literature review, and Table S1 gives basic demographic information on the interviewees. The interviewees gave written informed consent for anonymous quotations to be published.

Through this approach, I was able to identify a range of reported success factors. My proposed framework places these success factors into six categories, representing different components of the scaling up process:

- attributes of the specific tool or service being scaled up,
- attributes of the implementers,
- the chosen delivery strategy,
- attributes of the “adopting” community,
- the socio-political context, and
- the research context.

These categories were adapted from two previous typologies of scaling up (Box 1).

**Box 1. Two Previous Typologies of Scaling Up**

**Hanson and Colleagues' Typology of Constraints to Scaling Up**

Hanson and colleagues proposed that constraints to scaling up operate at five different levels: the community and household, health services delivery, health sector policy and strategic management, public policies cutting across sectors, and environmental and contextual characteristics [10].

**Simmons and Shiffman's “Elements of Scaling Up.”**

In their work on successfully scaling up reproductive health interventions, Simmons and Shiffman link the reproductive health innovation to “a resource team that promotes it; a user organization expected to adopt the innovation; a strategy to transfer it; and an environment in which the transfer takes place” [11].

My proposed framework differs from earlier scaling up frameworks (e.g., [10],[11]) in two ways. First, it draws upon insights from interviews with scale-up “leaders,” many of whom have led national or global health implementation programs. Second, it incorporates themes emerging from the recent literature (from 2007–2010).

## A Proposed Framework for Success

### Attributes of the Tool or Service Being Scaled Up

#### Simplicity

Keeping the intervention simple is widely considered to be a predictor of success [12]–[14]. The key to “rapid and massive scale-up” of antiretroviral therapy (ART) for people living with HIV in Malawi, say Harries and colleagues, was to “keep the principles and practices of ART delivery as simple as possible” [12]. Billings and colleagues argue that post-abortion care was successfully scaled up in Bolivia and Mexico because it met the criteria for scalability—cost-effectiveness, simplicity, and replicability [13]. A World Bank review of scaling up rural development interventions found that strong efforts at simplifying models or programs were associated with success [14].

One interviewee, who previously led a multilateral health organization, said: “If the intervention is simple, agreed, and there are no dissenting views, scale-up is much more likely to happen.”

#### Scientifically robust technical policies

Technical experts who have managed large-scale implementation argue that getting the technical policies scientifically robust before going to scale was crucial for success [15]. For example, before directly observed therapy short course (DOTs), a treatment program for tuberculosis, was scaled up across India, “all technical policies and detailed training modules for every level of staff were written, extensively revised, field tested over a period of several years, finalized, and disseminated widely” [15].

### Attributes of the Implementers

#### Strong leadership and governance

Case studies in the literature and interviewees' experiences of successful scale-up suggest that strong leadership played an important role [5],[16]. For example, a leadership development program in rural Egypt was associated with an increase in the number of new family planning, prenatal, and postnatal visits and a fall in the maternal mortality rate [16].

Several interviewees pointed to the important role of leadership in Uganda's success in scaling up HIV interventions [17]. The head of an African medical school argued: “Leadership is critical—at national level, regional level. Uganda's success in scale-up was because there was committed leadership to scaling up.”

#### Engaging local implementers and other stakeholders

A recurring theme among those I interviewed was the importance of getting buy-in from local implementers and other key stakeholders. The former director of a global health initiative (GHI) highlighted the role of local medical associations: “In neonatal health, if we don't have pediatric associations on board, forget it! For oral rehydration solution, after it was unequivocally demonstrated that it saved lives, the WHO and UNICEF were still doing local trials, randomized controlled trials. These didn't have *scientific* value but it led to buy-in locally.”

#### Using both state and non-state actors as implementers

Non-governmental organizations (NGOs) have played a crucial role in successful scale-up in many settings [5],[6]. A professor of global health said that an NGO in Bangladesh, the Bangladesh Rural Advancement Committee, is now reaching more people with health interventions than the government is reaching. A former director of a GHI discussed how non-governmental recipients of support from the Global Fund to Fight AIDS, Tuberculosis and Malaria have performed better than governmental recipients: “They [NGOs] are more nimble, there may be less corruption, they're quicker off their feet, individuals [in NGOs] are more motivated. From 2005, there are compelling data to show non-governmental recipients did better.”

Interviewees also discussed the role that private providers can play in scaling up, complementing the role of state actors. An expert in health systems reform discussed the importance of removing barriers in both sectors: “For a market solution: are you allowing entry to the market? For the public sector: are you getting tied up in bureaucracy?”

### The Chosen Delivery Strategy

#### Applying diffusion and social network theories

Rogers identified five factors that are positively associated with the faster diffusion of an innovation (Table 1), five types of adopters (innovators, early adopters, early majority, late majority, and laggards), and five stages in the adoption of a new innovation (awareness, interest, evaluation, trial, and adoption) [18]. Applying such diffusion theories at the right time, in the right place, within a health system has been a factor in the success of several LMIC scale-up programs [5],[19],[20]. McCannon and colleagues argue that successful scale-up programs have also paid attention to “the nature of the social network into which they wish to disseminate new practices,” for example, by considering how people interact and who are the early adopters [5].

**Table 1:** Factors associated with faster diffusion of an innovation.

Factor Associated with Faster Diffusion	Explanation
Relative advantage	Innovation addresses needs of adopter
Compatibility	Innovation is compatible with belief systems of adopter
Simplicity	Adopter finds the innovation simple
Trialability	Adopter has the opportunity to try the innovation before adopting it
Observability	Innovation and its results are observable by the adopter

Adapted from [18].

#### Cascade and phased approaches to scale-up

A national scale-up program for adolescent reproductive health services in health facilities and schools in Tanzania used a “cascade model”—regional trainers supervised district trainers, who in turn trained teachers and health workers [21]. The model succeeded in scaling up the intervention to 75%–100% of intended schools and health facilities in four districts of the country.

A related concept is the notion of going to scale in a phased manner, beginning with a pilot program, followed by step-wise expansion, learning lessons along the way to help refine further expansion [22]. Case studies of successful “phased” scale-up include Thailand's “100% condom” program [23], Guinea's national program to scale up HIV prevention and treatment tools [24], and scale-up of a harm reduction program in Asia [25].

#### Tailoring scale-up to the local situation, and decentralizing delivery

A variety of successful scale-up projects tailored implementation to local conditions on the ground and decentralized delivery so that clinics were closer to the target communities [11],[21],[26]. One interviewee, the expert in health systems reform, said: “You must tailor the message to local circumstances. You need some capacity—for example, a toolkit, a support network—to help local decision-makers to interpret a national decree.”

#### Adopting an integrated approach to scale-up

While many of the most high profile scale-up campaigns have been vertical in nature (e.g., scaling up insecticide-treated bed nets or ART), a complementary theme in the literature on successful implementation is the value of integrating scale-up activities into *existing* health systems [24],[27]. Using existing systems can be valuable if rapid scaling up is important, such as using established food and beverage delivery systems to rapidly distribute condoms [27]. HIV and mental health services have been scaled up in LMICs by integrating them into existing health services [26],[28]; similar integration is being planned for cancer services [29].

A related approach is to deliver disease control tools in an integrated manner, such as integrating child survival strategies with immunization [30], linking HIV and tuberculosis services [31], and integrating the control of different neglected tropical diseases [32].

### Attributes of the “Adopting” Community

#### An engaged, “activated” community

Scaling up by engaging community members or community health workers is well described in the literature. Examples include the Bangladesh Rural Advancement Committee's engagement of itinerant health workers [5] and Pakistan's “Lady Health Workers” program [33]. The active participation of the community in planning, implementing, and monitoring interventions is widely cited as a crucial factor in successful scale-up [11],[21],[24],[34].

A recent multicenter study found that a community-directed intervention strategy was more successful than other delivery strategies for scaling up distribution of ivermectin (an antiparasitic medication), vitamin A supplements, and insecticide-treated bed nets [34]. Recent evaluations of community health worker programs have shown a positive impact on child health [35], while a recent randomized trial in Bangladesh found that scaling up community-based participatory women's groups was associated with reduced neonatal mortality [36]. One interviewee, a former director of a GHI, pointed out that community engagement also means engaging *patients*: “If patients are not involved, you can't implement. In AIDS, it's the rule. Patients are experienced experts. They've gone through it. They *know*.”

### Socio-Political Context

#### Political will and national policies

National, regional, and local policy commitment are cited as important factors in the successful scale-up of breastfeeding in many LMICs (e.g., Bolivia and Madagascar) [37], post-abortion care in Guatemala [38], and DOTs in India [15]. Several interviewees said that political will has played a crucial role in scaling up adult male circumcision in some countries (e.g., Kenya), condom distribution in Thailand, and needle exchange in Iran. “Uganda has not been successful in scale-up of male circumcision,” said the head of an African medical school, “because there's no national policy yet. WHO recommends it should be scaled up. Therefore a policy or guideline must be in place—that's a success factor. Countries with a policy are making greater strides.”

#### Country ownership

Several interviewees discussed the importance of country ownership—and of moving away from traditional donor-recipient relationships in which donors dictate the terms—in the success of national scale-up programs in Africa. A former GHI director said that countries that had demonstrated successful scale-up through support from the Global Fund to Fight AIDS, Tuberculosis and Malaria were “special countries where the antipathy for neo-colonial relationships, the national pride, the desire for a new relationship between poor and rich countries overwhelmed the comfort with old, cozy donor relationships.”

### Research Context

#### Incorporating research into implementation (“learning and doing”)

Simmons and Shiffman argue that successful scale-up “requires the systematic use of evidence to guide the process and incorporate new learning” [11]. India's successful program to scale up DOTs adopted such an approach—monitoring was accompanied by timely feedback to implementers. Incorporating research into scale-up is also important for testing the transferability of a successful pilot program to a different setting [39], and for developing a “clearer understanding of the determinants of successful scaling-up” [11].

Several interviewees suggested that scale-up is more likely through synchronous implementation and research, which Peters and colleagues call “learning and doing” [40]. “Using data and experimenting underlies a lot of successful scale-up approaches,” said one interviewee, who leads health service scale-up projects in LMICs. “Mapping constraints, having the flexibility to redesign, learn-do cycling, being able to call in a more complete set of stakeholders—these kinds of approaches are more likely to lead to success.”

## Conclusions

By organizing success factors into a framework involving different components of the scaling up process, four key conclusions can be drawn.

First, a key lesson learned from successful scale-up efforts is that large-scale implementation is more likely if the intervention being scaled up is simple and technically sound and there is widespread consensus about its value. An important avenue of implementation research is therefore to simplify delivery—a good example is the landmark Development of Antiretroviral Therapy (DART) trial, which showed that ART can be safely delivered without complex and costly laboratory monitoring [41].

Second, the chances of success are likely to be increased by strong leadership and governance and the active engagement of a broad range of implementers, including non-state actors. Based on their experiences of successful scale-up, interviewees emphasized that GHIs are likely to fail unless they engage *local* implementers and the recipient community itself, an assertion supported by a growing body of research evidence.

Third, there is no single or straightforward delivery strategy that offers a formula for success. As Gilson and Schneider say, “there is no simple recipe for managing scaling-up processes” [42]. The framework laid out in this Essay suggests that a wide variety of different strategies could all have an impact. Empirical research will help to define which strategy is best suited to a particular health challenge and setting. Such research will also help implementers to better understand the complex array of contextual factors, such as politics, socio-cultural norms and beliefs, and the fiscal environment, that can influence scale-up success.

Finally, the field of implementation science in LMICs would be advanced if scale-up efforts were always accompanied by research [2]. Any scaling up process, say Hanson and colleagues, should “include both opportunities to learn through action and a way to feed the lessons of experience into strategies to strengthen implementation” [43].

## Supporting Information

Text S1 **Search strategy used for literature review.**
(DOCX)

Table S1 **Basic demographic information on interviewees.**
(DOCX)

## Abbreviations

ART: antiretroviral therapy
DOTs: directly observed therapy short course
GHI: global health initiative
LMIC: low- and middle-income country
NGO: non-governmental organization

## References

[1] Feachem RGA (2004). The research imperative: fighting AIDS, TB and malaria.. Trop Med Int Health.

[2] Madon T, Hofman KJ, Kupfer L, Glass RI (2007). Implementation science.. Science.

[3] Management Systems International (2006). Scaling up—from vision to large-scale change.. A management framework for practitioners.

[4] Bergh AM, van Rooyen E, Pattinson RC (2008). Scaling up kangaroo mother care in South Africa: ‘on-site’ versus ‘off-site’ educational facilitation.. Hum Resour Health.

[5] McCannon CJ, Berwick D, Massoud MR (2007). The science of large-scale change in global health.. JAMA.

[6] Mangham LJ, Hanson K (2010). Scaling up in international health: what are the issues?. Health Policy Plan.

[7] Yamey G (2010 October 5). Malaria in Africa: the net gains of keeping mosquitoes at bay.. The Telegraph.

[8] Yamey G (2010). Do schools hold the key to controlling parasitic disease?. BMJ.

[9] The PLoS Medicine (2009). Time for a “third wave” of malaria activism to tackle the drug stock-out crisis.. PLoS Med.

[10] Hanson K, Ranson MK, Oliviera-Cruz V, Mills A (2003). Expanding access to priority health interventions: a framework for understanding the constraints to scaling-up.. J Int Dev.

[11] Simmons R, Shiffman J, Simmons R, Fajans P, Ghiron L (2007). Scaling up health service innovations: a framework for action.. Scaling up health service delivery.

[12] Harries AD, Zachariah R, Jahn A, Schouten, Kamoto K (2009). Scaling up antiretroviral therapy in Malawi—implications for managing other chronic diseases in resource-limited countries.. J Acquir Immune Defic Syndr.

[13] Billings DL, Crane BB, Benson J, Solo J, Fetters T (2007). Scaling-up a public health innovation: a comparative study of post-abortion care in Bolivia and Mexico.. Soc Sci Med.

[14] World Bank (2003). Scaling up the impact of good practices in rural development: a working paper to support implementation of the World Bank's rural development strategy.. Report no. 26031.

[15] Khatri GR, Frieden TR (2002). Rapid DOTS expansion in India.. Bull World Health Organ.

[16] Mansour M, Mansour JB, El Swesy AH (2010). Scaling up proven public health interventions through a locally owned and sustained leadership development programme in rural Upper Egypt.. Hum Resour Health.

[17] Konde-Lule JK (1995). The declining HIV seroprevalence in Uganda: what evidence?. Health Transit Rev.

[18] Rogers E (1995). Diffusion of innovations..

[19] Fixsen DL, Naoom SF, Blase KA, Friedman RF, Wallace F (2005). Implementation research: a synthesis of the literature.. http://cfs.fmhi.usf.edu/pub-details.cfm?pubID=137.

[20] Larson CP, Saha UR, Nazrul H (2009). Impact monitoring of the national scale up of zinc treatment for childhood diarrhea in Bangladesh: repeat ecologic surveys.. PLoS Med.

[21] Renju J, Makokha M, Kato C, Medard L, Andrew B (2010). Partnering to proceed: scaling up adolescent sexual reproductive health programmes in Tanzania. Operational research into the factors that influenced local government uptake and implementation.. Health Res Policy Syst.

[22] Quelapio MID, Mira NRC, Orillaza-Chi RB, Belen V, Muñez N (2010). Responding to the multidrug-resistant tuberculosis crisis: mainstreaming programmatic management to the Philippine National Tuberculosis Programme.. Int J Tuberc Lung Dis.

[23] Rojanapithayakorn W, Hanenberg R (1996). The 100% condom program in Thailand.. AIDS.

[24] Binswanger HP (2000). Scaling up HIV/AIDS programs to national coverage.. Science.

[25] Chatterjee A, Sharma M (2010). Moving from a project to a programmatic response: scaling up harm reduction in Asia.. Int J Drug Policy.

[26] Kiima D, Jenkins R (2010). Mental health policy in Kenya—an integrated approach to scaling up equitable care for poor populations.. Int J Mental Health Systems.

[27] Watts C, Kumaranayake L (1999). Thinking big: scaling-up HIV-1 interventions in sub-Saharan Africa.. Lancet.

[28] Walton DA, Farmer P, Lambert W, Léandre F, Koenig SP (2004). Integrated HIV prevention and care strengthens primary health care: lessons from rural Haiti.. J Public Health Policy.

[29] CanTreat International (2010). Scaling up cancer diagnosis and treatment in developing countries: what can we learn from the HIV/AIDS epidemic?. Ann Oncol.

[30] Clements CJ, Nshimirimanda D, Gasasira A (2008). Using immunization delivery strategies to accelerate progress in Africa towards achieving the Millennium Development Goals.. Vaccine.

[31] Nsutebu EF, Walley JD, Mataka E, Simon CF (2001). Scaling-up HIV/AIDS and TB home-based care: lessons from Zambia.. Health Policy Plan.

[32] Hotez PJ, Molyneux DH, Fenwick A, Ottesen E, Sachs SE (2006). Incorporating a rapid-impact package for neglected tropical diseases with programs for HIV/AIDS, tuberculosis, and malaria.. PLoS Med.

[33] Bhutta Z Memon ZA, Soofi S, Salat MS, Cousens S (2008). Implementing community-based perinatal care: results from a pilot study in rural Pakistan.. Bull World Health Organ.

[34] CDI Study Group (2010). Community-directed interventions for priority health problems in Africa: results of a multicountry study.. Bull World Health Organ.

[35] Haines A, Sanders D, Lehmann U, Rowe AK, Lawn JE (2007). Achieving child survival goals: potential contribution of community health workers.. Lancet.

[36] Azad K, Barnett S, Banerjee B, Shaha S, Khan K (2010). Effect of scaling up women's groups on birth outcomes in three rural districts in Bangladesh: a cluster-randomised controlled trial.. Lancet.

[37] Bhandari N, Kabir AK, Salam MA (2008). Mainstreaming nutrition into maternal and child health programmes: scaling up of exclusive breastfeeding.. Matern Child Nutr.

[38] Kestler E, Valencia L, Del Valle V, Silva A (2006). Scaling up post-abortion care in Guatemala: initial successes at national level.. Reprod Health Matters.

[39] Sim F, Mackie P (2008). Scaling up training and education for the health workforce—international collaboration.. Public Health.

[40] Peters DH, El-Saharty S, Janovsky K, Peters D, El-Saharty S, Siadat B, Janovsky K, Vujicic M (2009). From evidence to learning and action.. Improving health service delivery in developing countries.

[41] DART Trial Team (2010). Routine versus clinically driven laboratory monitoring of HIV antiretroviral therapy in Africa (DART): a randomised non-inferiority trial.. Lancet.

[42] Gilson L, Schneider H (2010). Managing scaling up: what are the key issues?. Health Policy Plan.

[43] Hanson K, Cleary S, Schneider H, Tantivess S, Gilson S (2010). Scaling up health policies and services in low- and middle-income settings.. BMC Health Serv Res.

