# Supplementary material for: Scaling Up Global Health Interventions: A Proposed Framework for Success
Source: PLoS Med. 2011 Jun 28;8(6):e1001049. doi: 10.1371/journal.pmed.1001049 (PMC3125181; doi:10.1371/journal.pmed.1001049)
Supplement: Text S1 — Search strategy used for literature review. (DOCX) [file pmed.1001049.s001.docx]

**Text S1: Search Strategy Used for Literature Review**

**(a) PubMed Search**

I began my search using PubMed [1]. The term “implementation science” is a recent one—the first time that it appears in the title or abstract of a paper archived in PubMed is 2006 [2]. Thus the use of this term alone would have missed several important papers on implementing health tools or services to scale in LMICs. Therefore I also used the search terms “scaling up” or “scaling-up,” which have been widely used in the health literature since 2001 [3]. Mangham and Hanson argue that “although ‘scaling up’ is now frequently used in the international health literature, there have been few attempts to define what is meant by the term” [3]. The term is primarily used, they say, to “describe the ambition or process of expanding the coverage of health interventions” [3], a working definition that I used in my study.

I searched PubMed for articles that contained the term “implementation science” in the title or abstract, or the terms “scaling up” or “scaling-up” in the title (my search strategy was “implementation science[tiab] OR scaling up[ti] OR scaling-up[ti]”). My PubMed search strategy retrieved 290 articles. After reviewing the titles and abstracts of these 290 articles, I retrieved 93 full text articles that met my inclusion criteria (and that were freely available in the University of California, San Francisco library). Having read the full versions of these 93 articles, I excluded 43 of these because they were not directly relevant to this Essay. Thus I included 50 articles from my PubMed search in my literature review.

**(b) Web of Science search**

I initially used a similar strategy to search Web of Science [4]. My first search used the search term “implementation science” in the topic, and also “scaling up” or “scaling-up” in the title (i.e. my search strategy was: “implementation science” in the topic OR “scaling up” in the title OR “scaling-up” in the title). This retrieved 38,776 articles [“set 1”], the vast majority of which were unrelated to health. I thus narrowed the search through a two-stage process. First, I searched for articles containing “global health” or “international health” anywhere in the article (“global health” in the topic OR “international health” in the topic), which yielded 44,632 results [“set 2”]. Second, I combined sets 1 and 2 using the Boolean operator AND (“set 1 AND set 2”), which yielded 217 results. I reviewed the titles and abstracts of these 217 articles, and retrieved 43 full text articles that met the inclusion criteria (and that were freely available in the UCSF library). Of these 43 articles, 23 had been identified by my PubMed search, leaving 20 additional full text articles. Having read the full versions of these 20 articles, I excluded 11 of these because they were not directly relevant to this Essay. Thus I included 9 articles from my Web of Science search in my literature review.

**(c) Additional articles**

Finally, I identified an additional 84 relevant articles from: the reference lists of papers identified by my PubMed and Web of Science searches; asking interviewees to suggest the most important papers in the field; and from two special journal issues on scaling up (*Health Policy and Planning* in March 2010 [5], and *BMC Health Services Research* in July 2010 [6]). Thus I read a total of 143 articles full text articles. The flow chart below summarizes the retrieval of articles.


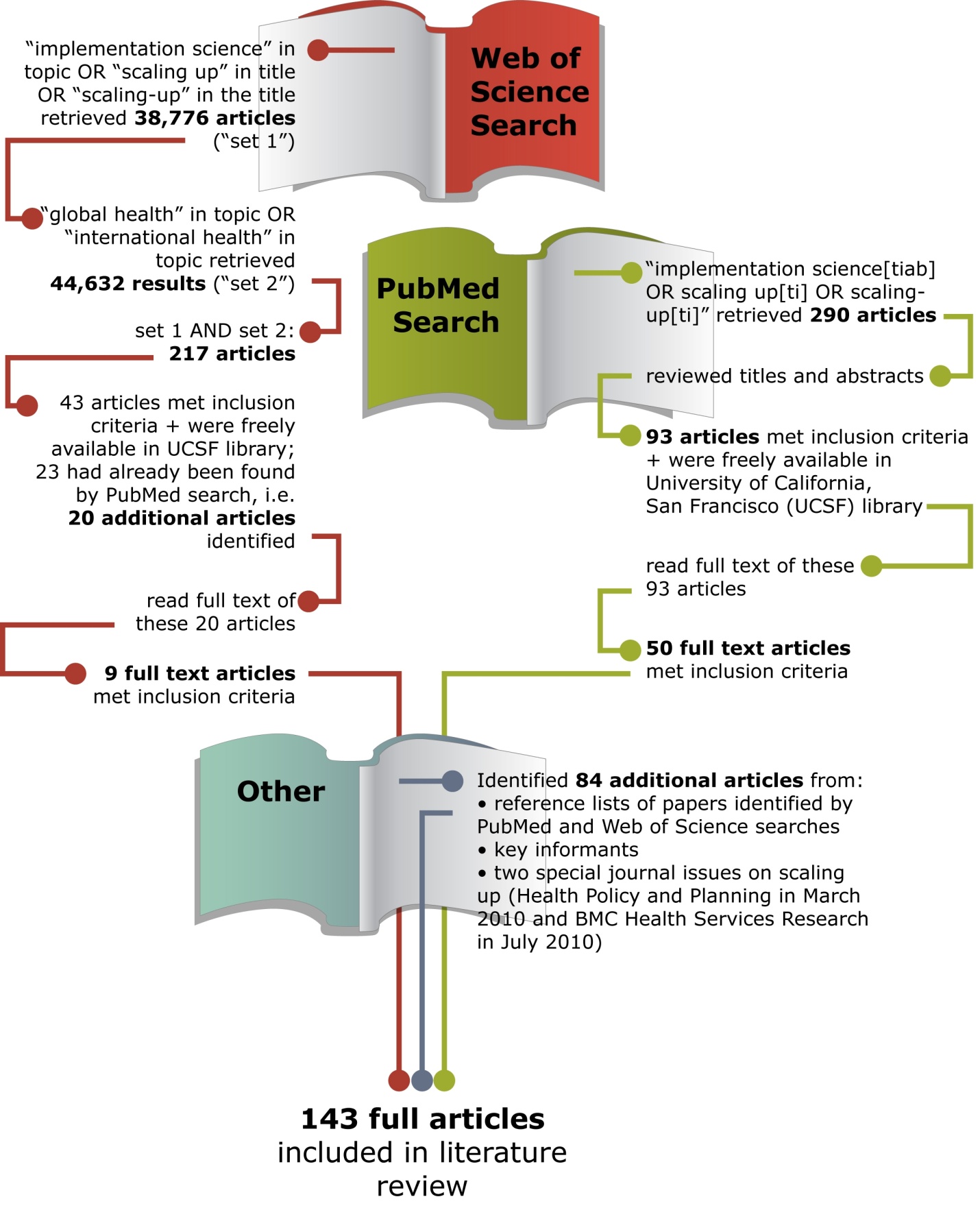


**References**

PubMed.gov. Available: <http://www.ncbi.nlm.nih.gov/pubmed> Accessed: 23 January 2011

[Rubenstein LV](http://www.ncbi.nlm.nih.gov/pubmed?term=%22Rubenstein%20LV%22%5BAuthor%5D), [Pugh J](http://www.ncbi.nlm.nih.gov/pubmed?term=%22Pugh%20J%22%5BAuthor%5D) (2006). Strategies for promoting organizational and practice change by advancing implementation research. [*J Gen Intern Med*](javascript:AL_get(this,%20'jour',%20'J%20Gen%20Intern%20Med.'););21 Suppl 2:S58-64.

Mangham LJ, Hanson K (2010). Scaling up in international health: what are the issues? *Health Policy Plan* 25:85-96.

Web of Science. Available: <http://thomsonreuters.com/products_services/science/science_products/a-z/web_of_science>. Accessed: 23 January 2011

*Health Policy and Planning* V**olume 25, Number 2, March 2010. Available:** [http://heapol.oxfordjournals.org/content/vol25/issue2](http://heapol.oxfordjournals.org/content/vol25/issue2/). **Accessed** 23 January 2011

Hanson K, Cleary S, Schneider H, Tantivess S, Gilson L (eds) (2010). Scaling-up health services in low- and middle-income settings. *BMC Health Services Research* Volume 10, supplement 1, July 2010. Available: <http://www.biomedcentral.com/1472-6963/10?issue=S1>. **Accessed** 23 January 2011
