# Supplementary material for: Scaling Up Global Health Interventions: A Proposed Framework for Success
Source: PLoS Med. 2011 Jun 28;8(6):e1001049. doi: 10.1371/journal.pmed.1001049 (PMC3125181; doi:10.1371/journal.pmed.1001049)
Supplement: Table S1 — Basic demographic information on interviewees. (DOCX) [file pmed.1001049.s002.docx]

**Table S1. Basic Demographic Information on Interviewees**

| **Interviewee** | **Current Position** | | **Previous Experience** |
| --- | --- | --- | --- |
| Interviewee # 1 | Academic global health post in a high income country (HIC); university leadership post | | Leadership of a multilateral organization; has led large-scale implementation in low- and middle-income countries (LMICs); research on communicable diseases |
| Interviewee # 2 | Academic global health post in a HIC; university leadership post | | Previous academic posts; leadership positions in multilateral organizations; has led large-scale implementation in LMICs; public health research |
| Interviewee # 3 | Academic global health post in a HIC; leads health service scale-up projects in LMICs | | Research on scaling up in LMICs with a focus on health systems |
| Interviewee # 4 | Academic public health post in a MIC in Africa; advises national government on large-scale implementation | | Research on scaling up in LMICs, with a focus on building research capacity and on health systems |
| Interviewee # 5 | Academic health policy post in a HIC; specializes in health systems reform | | Research on health systems reform in both LMICs and HICs |
| Interviewee # 6 | Academic global health post in a HIC; university leadership post | | Research on large-scale implementation with a focus on non-communicable diseases; previous senior positions in national public health service |
| Interviewee # 7 | Academic post in global health in a HIC; university leadership post | | Academic leadership positions at global health agencies |
| Interviewee # 8 | Academic public health post; principal of a medical school in a LIC in Africa | Led national scale-up campaigns addressing communicable diseases | |
| Interviewee # 9 | Academic public health post at a school of global health in a HIC | Research on large-scale implementation, with a focus on communicable diseases; previous senior positions in regional public health service | |
| Interviewee # 10 | Women’s health researcher in a MIC in Latin America | Research on implementing women’s health interventions | |
| Interviewee # 11 | Implementation science researcher in a HIC | Academic public health posts, with a focus on implementation of health interventions in a HIC | |
| Interviewee # 12 | Director a scale-up program in a LIC | Background in clinical medicine and global public health, dividing time between a LIC and a HIC | |
| Interviewee # 13 | Professor of epidemiology, with a focus on global public health | Clinical trials, and public health research related to women’s and children’s health | |
| Interviewee # 14 | Senior leadership position at an NGO that manages large-scale implementation LMICs | Past background in business and technology | |
